# Curcumin is a promising inhibitor of genotype 2 porcine reproductive and respiratory syndrome virus infection

**DOI:** 10.1186/s12917-017-1218-x

**Published:** 2017-10-10

**Authors:** Taofeng Du, Yunpeng Shi, Shuqi Xiao, Na Li, Qin Zhao, Angke Zhang, Yuchen Nan, Yang Mu, Yani Sun, Chunyan Wu, Hongtao Zhang, En-Min Zhou

**Affiliations:** 1 Department of Preventive Veterinary Medicine, College of Veterinary Medicine, Northwest A&F University, Yangling, Shaanxi 712100 China; 2 Experimental Station of Veterinary Pharmacology and Veterinary Biotechnology, Ministry of Agriculture, Yangling, Shaanxi 712100 China; 3 Department of Pathology and Lab Medicine, University of Pennsylvania Perelman School of Medicine, 3620 Hamilton Walk, Philadelphia, PA 19104 USA

**Keywords:** PRRSV, Curcumin, Binding, Internalization, Virus-mediated cell fusion

## Abstract

**Background:**

Porcine reproductive and respiratory syndrome virus (PRRSV) could lead to pandemic diseases and huge financial losses to the swine industry worldwide. Curcumin, a natural compound, has been reported to serve as an entry inhibitor of hepatitis C virus, chikungunya virus and vesicular stomatitis virus. In this study, we investigated the potential effect of curcumin on early stages of PRRSV infection.

**Results:**

Curcumin inhibited infection of Marc-145 cells and porcine alveolar macrophages (PAMs) by four different genotype 2 PRRSV strains, but had no effect on the levels of major PRRSV receptor proteins on Marc-145 cells and PAMs or on PRRSV binding to Marc-145 cells. However, curcumin did block two steps of the PRRSV infection process: virus internalization and virus-mediated cell fusion.

**Conclusions:**

Our results suggested that an inhibition of genotype 2 PRRSV infection by curcumin is virus strain-independent, and mainly inhibited by virus internalization and cell fusion mediated by virus. Collectively, these results demonstrate that curcumin holds promise as a new anti-PRRSV drug.

## Background

Porcine reproductive and respiratory syndrome (PRRS), a serious pandemic disease, causes huge economic losses to the swine industry. Clinically, PRRS causes fetal abortion in pregnant sows and respiratory disease in pigs of all ages [1, 2]. The etiological agent, PRRS virus (PRRSV), is an enveloped single-stranded positive-sense RNA virus belonging to the *Arteriviridae* family [3]. PRRSV strains belong to two major genotypes, the European (type 1) and North American (type 2) genotypes, with distinct genetic profiles and geographic distributions [4–6]. Highly pathogenic PRRSV (HP-PRRSV) isolates from China belong to the type 2 genotypic group [7].

Although PRRSV was discovered over twenty years ago, an effective vaccine still has not been developed for the following reasons: (1) Infection of pigs can occur during every phase of production; (2) The semen of boars is a hiding place for virus; (3) The virus is passed between pig farms through multiple routes; (4) High virus mutation frequency is observed; (5) Persistence of infection is observed; (6) Immunosuppression induced by infection prevents virus control [8, 9]; and (7) Although, attenuated PRRSV vaccines can induce weak humoral and cell-mediated immune responses, but have less protection against heterologous strains [10]. Therefore, development of new antiviral drugs or vaccine adjuvants is needed to control PRRSV.

Curcumin, 1,7-bis(4-hydroxy-3-methoxyphenyl)-1,6-heptadiene-3,5-dione), also known as diferuloylmethane, is a natural bioactive polyphenolic compound isolated from *Curcuma longa* rhizomes. The main components of *C. longa* include diferuloylmethane (65–80%), demethoxycurcumin (15–25%) and bisdemethoxycurcumin (5–15%). Curcumin accounts for most of the biological and pharmacological effects of *C. longa* including immunomodulating, anti-tumor, anti-inflammatory, antioxidant, antimutagenic, antibacterial, antifungal and antiviral activities [11–14]. Notably, curcumin inhibits entry of several types of viruses into cells, including hepatitis C virus (HCV), chikungunya virus (CHIKV) and vesicular stomatitis virus (VSV) [13, 15]. To our knowledge, no potential anti-PRRSV effect of curcumin has yet been reported. Therefore, we evaluated curcumin as an anti-PRRSV agent in Marc-145 cells and PAM and found it inhibited PRRSV infection by preventing virus internalization and virus-mediated cell fusion.

## Methods

### Viruses, cells and compounds

Four North American PRRSV strains were tested, including highly pathogenic strains PRRSV GD-HD (HP-PRRSV/GD-HD) (GenBank ID: **KP793736**), HP-PRRSV/JXA1 (GenBank ID: **EF112445**), classic PRRSV VR-2332 (GenBank ID: **EF442771**) and a low pathogenic strain, PRRSV CH-1a (GenBank ID: **AY032626**). All strains were propagated and titrated in Marc-145 cells grown in Dulbecco’s modified Eagle’s medium (DMEM) (Gibco, USA) supplemented with 3% heat-inactivated fetal bovine serum (HI-FBS; Biological Industries, CT, USA). Marc-145 cells were cultured in DMEM with 10% HI-FBS, 100 units/ml penicillin and 100 μg/ml streptomycin (Gibco) at 37 °C in 5% CO_2_ incubator. PAMs were obtained from healthy 6-week-old PRRSV-negative pigs using modified lung lavage collection method as previously described [16]. PAMs were maintained in RPMI-1640 medium (Gibco) supplemented with 10% HI-FBS and 1% penicillin-streptomycin-amphotericin B (100×) (Life Technologies Corp., CA, USA). Curcumin (Sigma-Aldrich, St. Louis, MO, USA) was dissolved in dimethyl sulphoxide (DMSO) to make 5, 10, and 15 mM/L as the stock solution and was used at 1/1000 dilution.

### Cell viability assay

To evaluate curcumin cytotoxicity, a Cell Counting Kit-8 (CCK8) (Sigma-Aldrich) was used [17]. Briefly, Marc-145 cells and PAMs were seeded into 96-well plates at densities of 1 × 10^4^/well or 1 × 10^5^/well in the presence of various curcumin concentrations for 48 h. 10 μL/well of CCK-8 reagent was added before incubation for 2 h. Absorbance was measured at 450 nm.

### Virus inhibition assay

To examine curcumin antiviral activity during early infection stages, Marc-145 cells or PAMs were treated with various curcumin concentrations at 37 °C for 1 h before, during or after GD-HD infection at a multiplicity of infection (MOI: 0.1). The inoculum was discarded and cells were washed three times with PBS and maintained in DMEM containing 3% HI-FBS at 37 °C. After incubation for 36 h, supernatants and cells were harvested to measure virus titers, PRRSV N protein levels and ORF7 mRNA levels. Chloroquine (20 μM) was used as the positive control.

### Virus binding assay

Marc-145 cells were seeded into 24-well plates, incubated for 24 h at 37 °C, then treated with curcumin using the following three distinct methods: (i) Virus pretreatment: The virus GD-HD strain (MOI: 100) was incubated with curcumin for 10 min at 37 °C and the mixture was cooled to 4 °C before infection. Then, Marc-145, cells, after prechilling at 4 °C for 1 h, were infected with pretreated virus at 4 °C for 1 h. (ii) Cell pretreatment: Marc-145 cells were treated with curcumin for 1 h at 37 °C and GD-HD was added to cells for 1 h at 4 °C. (iii) Co-treatment: Marc-145 cells were prechilled at 4 °C for 1 h and infected with GD-HD in the presence of curcumin at 4 °C for 1 h. After incubation, cells were washed with ice-cold PBS and harvested to detect PRRSV ORF7 gene using qRT-PCR.

### Virus internalization assay

Marc-145 cells were prechilled for 1 h at 4 °C then incubated for 1 h at 4 °C with GD-HD (MOI: 0.1). After virus attachment, cells were washed three times with ice-cold PBS and incubated at 37 °C for 1 h in DMEM with curcumin or chloroquine. Next, cells were washed three times with PBS and incubated with proteinase K (Sigma) for 45 min at 4 °C to get rid of non-internalized viruses. A cocktail (Roche, Meylan, France) was used to inactivate proteinase K. And then the cells were washed thrice with PBS plus 0.2% BSA and maintained in medium without curcumin at 37 °C. At 36 h post infection, supernatants and cells were harvested to measure virus titers and PRRSV N protein levels.

### Virus post-internalization assay

After internalization of GD-HD (MOI: 0.1), Marc-145 cells were washed three times with PBS and treated with curcumin in DMEM containing 3% HI-FBS. After incubation at 37 °C for 36 h, culture medium and cells were harvested to measure virus titers and PRRSV N protein levels, respectively. Chloroquine (20 μM) was used as the positive control. Meanwhile, a low pH-dependent cell fusion assay was used to examine whether curcumin inhibited virus-mediated cell fusion in PRRSV-infected Marc-145 cells [18]. Briefly, Marc-145 cells were infected with GD-HD (MOI: 5) at 37 °C. After incubation for 36 h, cells were treated with curcumin at 37 °C for 2 h, washed with PBS, then incubated with fusion medium [10 mM Na_2_HPO_4_, 10 mM NaH_2_PO_4_, 150 mM NaCl, 10 mM 2-(N-morpholino) ethanesulfonic acid, adjusted to pH 5.6 with HCl] for 2 min at room temperature. Cells were washed after fusion medium was discarded, incubated with 3% DMEM for 2 h at 37 °C, then fixed with 70% ethanol, stained with fuchsine solution and viewed using light microscopy (Motic AE31, Xiamen, China).

### Quantitative reverse transcription-PCR (qRT-PCR)

Total RNA was extracted from Marc-145 cells using Total RNA Kit (OMEGA Bio-Tek, GA, USA) using the manufacturer’s instructions. Reverse transcription of RNA to cDNA was performed using a Primescript RT reagent Kit (TaKaRa, Dalian, China). Reverse-transcribed products were analyzed using a StepOnePlus Real-Time PCR System (Applied Biosystems, CA, USA) and FastStart Universal SYBR Green Master (Roche, Basel, Switzerland). Specific primer sequences were as follows: Forward primer, PRRSV ORF-7: 5′-AGATCATCATCGCCCAACAAAAC-3′ and Reverse primer, PRRSV ORF-7: 5′-GACACAATTGCCGCTCACTA-3′; Forward primer, monkey β-actin: 5′-TCCCTGGAGAAGAGCTACGA-3′ and Reverse primer, monkey β-actin: 5′-AGCACTGTGTTGGCGTACAG-3′. β-actin was used as an internal control.

### Western blot

Cells were lysed with NP-40 lysis buffer containing 1 mM PMSF (Beyotime, Beijing, China). Whole cell lysates were electrophoresed on 12% glycine SDS-PAGE gels, transferred to PVDF membranes, and membranes were incubated with one of the following primary antibodies: mouse anti-PRRSV-N monoclonal antibody 6D10 (1 μg/ml, produced in our laboratory), mouse anti-non-muscle myosin heavy chain II-A monoclonal anti-idiotypic antibody Mab2-5G2 [19], mouse anti-CD163 polyclonal antibody (1:3000, produced in our laboratory), anti-vimentin (Santa Cruz Biotechnology, CA, USA), rabbit anti-CD169 polyclonal antibody (Proteintech Group, Inc., China) or anti-α-tubulin antibody (Sigma) at dilutions recommended by the manufacturers. HRP-conjugated goat anti-mouse IgG or HRP-conjugated goat anti-rabbit IgG (1:2000, Jackson Labs, ME, USA) was used as secondary antibody. Protein signals were visualized using ECL reagent (Pierce, Rockford, IL, USA).

### Virus titration

Virus progeny were titrated as described previously [20]. Briefly, Marc-145 cells were seeded into 96-well plates, incubated for 24 h at 37 °C then incubated with 100 μL/well of 10-fold serial dilutions of virus supernatants. After a 6-day incubation, the 50% cell culture infection dose (CCID_50_) was calculated using the Reed-Muench method.

### Statistical analysis

All experiments were performed independently at least three times. Western blots were analyzed using Image Lab 5.1 software (Bio-Rad Laboratories Inc., CA, USA). Virus titers and virus RNA levels were analyzed using GraphPad Prism software (GraphPad Software Inc., San Diego, CA, USA). Statistical significance was determined using Student’s t-test for comparison of two groups or by one-way analysis of variance (ANOVA) for comparison of more than two groups. A value of *P* < 0.05 was considered statistically significant.

## Results

### Curcumin treatment inhibited PRRSV infection of Marc-145 cells

**Fig. 1:**
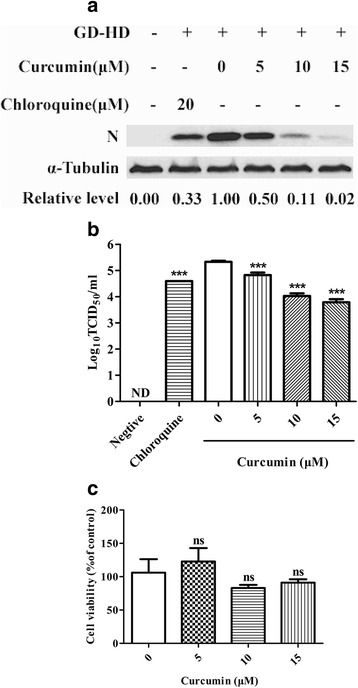
Effects of curcumin on PRRSV infection of Marc-145 cells. Marc-145 Cells were co-incubated with GD-HD (MOI: 0.1) and various curcumin concentrations at 37 °C for 1 h. Cells were washed and incubated at 37 °C for 36 h in medium without curcumin. PRRSV N protein expression levels were analyzed using western blot with α-tubulin as a control for sample loading (**a**). Virus titers in the supernatants were determined using CCID_50_ (**b**). Curcumin cytotoxicity was determined in Marc-145 cells using a CCK8 assay and is presented as experimental cell viability relative to cell viability of untreated control (100%) (**c**). Each value represents the mean ± SD from three independent experiments (*, *p* < 0.05; **, *p* < 0.01; ***, *p* < 0.001; ND means non-detectable)

The ability of curcumin to inhibit PRRSV infection was evaluated in Marc-145 cells by detection of PRRSV N protein and virus production. Chloroquine served as the positive control by inhibiting the necessary initial stage of virus infection, endosome acidification, in a pH-dependent manner [21]. Co-incubation of Marc-145 cells for 1 h with curcumin and PRRSV (GD-HD) significantly reduced N protein (Fig. 1a) and PRRSV progeny titers (Fig. 1b) and 5 to 15 μM curcumin reduced N protein levels in a dose-dependent manner from 50 to 98%, respectively. Moreover, using 15 μM curcumin, virus titers deceased by 1.55 log relative to supernatants with PRRSV alone. CCK-8 testing of Marc-145 cells with added curcumin indicated that curcumin exhibited no significant cytotoxic effects (Fig. 1c).

### Curcumin inhibited the infection of different genotype 2 PRRSV strains

**Fig. 2:**
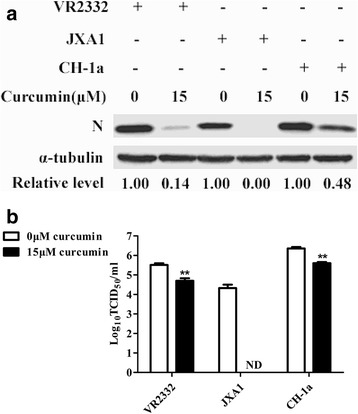
Assessment of virus strain-dependence of curcumin anti-PRRSV activity. Marc-145 cells were co-treated with PRRSV (MOI: 0.1) and 15 μM curcumin at 37 °C for 1 h. Cells were washed and incubated in medium without curcumin at 37 °C for 36 h. N protein (**a**) and PRRSV production (**b**) were analyzed by western blot and virus titration assay, respectively. Each value represents the mean ± SD from three independent experiments (*, *p* < 0.05; **, *p* < 0.01; ***, *p* < 0.001; ND means non-detectable)

To assess whether curcumin inhibits infection of Marc-145 cells by various genotype 2 PRRSV strains, classic strains PRRSV VR-2332 and HP-PRRSV/JXA1, and a low pathogenic strain, PRRSV CH-1a, were studied in the same way as the pathogenic GD-HD strain described above. Using 15 μM curcumin, relative N protein levels for VR-2332, JXA1 and CH-1a were 0.14, 0.00 and 0.48, respectively, as compared to PRRSV-only control (Fig. 2). Likewise, virus titers of curcumin-treated groups were decreased by varying log values (VR2332, 0.54 log; JXA1, 4.32 log; CH-1a, 0.75 log) vs. PRRSV-only controls (Fig. 2b).

### Curcumin had no effect on PRRSV receptor expression, but inhibited PRRSV infection during the inoculation phase of virus infection

**Fig. 3:**
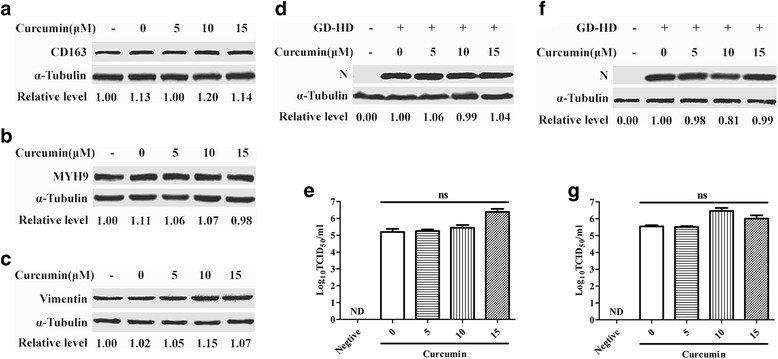
PRRSV receptors and infectivity analysis of Marc-145 cells treated with curcumin before or after infection. Marc-145 cells were incubated with various curcumin concentrations at 37 °C for 1 h. CD163 (**a**), MYH9 (**b**) and vimentin (**c**) protein levels were measured by western blot. Marc-145 cells were incubated with curcumin before addition of GD-HD (MOI: 0.1, **d**, **e**) or after infection (**f**, **g**) with indicated curcumin concentrations. After incubation at 37 °C for 1 h, cells were maintained in medium without curcumin for 36 h. Next, PRRSV N protein expression levels (**d** and **f**) and viral production (**e** and **g**) were determined by western blot and virus titration assay, respectively. Each value represents the mean ± SD from three independent experiments (*, *p* < 0.05; **, *p* < 0.01; ***, *p* < 0.001; ND means non-detectable)

To investigate whether curcumin downregulated known PRRSV receptors, CD163 [22], non-muscle myosin heavy chain 9 (MYH9) [19] and vimentin [23] expression levels in Marc-145 cells were tested using western blot analysis. Curcumin did not cause downregulation of cellular PRRSV receptors (Fig. 3a-c).

We next assessed curcumin antiviral effect upon addition to Marc-145 cells before or after PRRSV infection. As shown in Fig. 3d-g, N protein expression levels and progeny titers were not reduced by either of these treatment methods. Therefore, curcumin does not render Marc-145 cells more susceptible to PRRSV infection, but does interfere with PRRSV infection during the inoculation period of infection.

### Curcumin did not affect PRRSV binding to Marc-145 cells

**Fig. 4:**
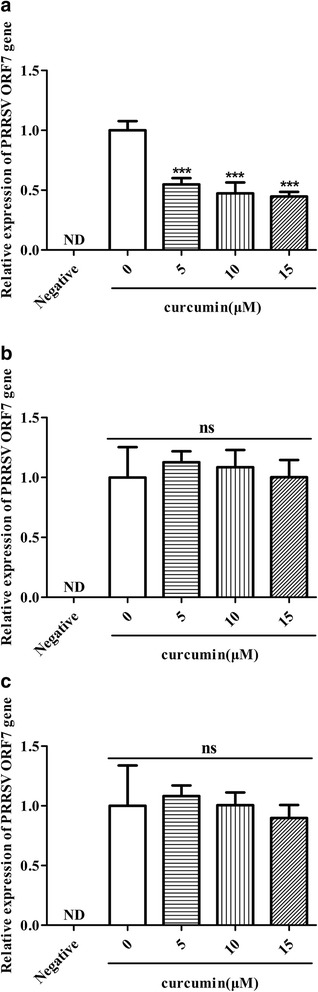
Measurement of a curcumin effect on PRRSV absorption in Marc-145 cells. Marc-145 cells were pre-chilled at 4 °C for 1 h followed by incubation with curcumin-pretreated GD-HD (MOI: 100) (**a**), or a mixture of GD-HD (MOI: 100) and curcumin (**c**) at 4 °C for 1 h. For pretreatment of cells, Marc-145 cells were pre-incubated with curcumin at 37 °C for 1 h. After incubating cells at 4 °C for 1 h, both GD-HD (MOI: 100) and curcumin were added and incubated for 1 h at 4 °C (**b**). After washing out unbound virus, total RNA was extracted from the cells and the relative cell-bound PRRSV RNA levels were measured using qRT-PCR

PRRSV binding to Marc-145 cells is considered a first step in infection. Thus, a virus binding assay was performed to determine if curcumin inhibits this binding. Virus RNA was collected and relative PRRSV ORF7 gene levels were analyzed using qRT-PCR. When virus was pretreated with curcumin, PRRSV ORF7 gene levels decreased in a dose-dependent manner (Fig. 4a). However, pretreatment of cells with curcumin or treatment with curcumin during virus adsorption did not affect PRRSV ORF7 gene levels (Fig. 4b, c). Therefore, curcumin has the potential to affect the infectivity of PRRSV at 37 °C, but did not inhibit virus attachment to Marc-145 cells.

### Curcumin inhibited PRRSV internalization

**Fig. 5:**
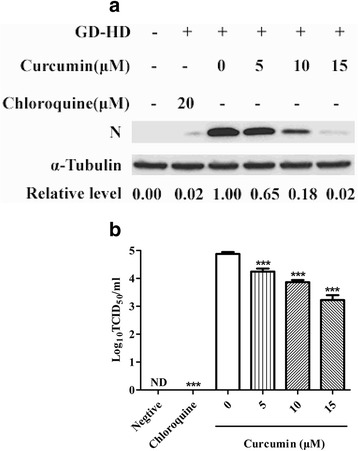
Analysis of curcumin treatment effect on PRRSV internalization in Marc-145 cells. Pre-chilled Marc-145 cells were infected with GD-HD (MOI: 0.1) at 4 °C for 1 h then incubated with indicated curcumin concentrations at 37 °C for 1 h. After incubation, non-internalized viruses were removed and maintained in medium without curcumin for 36 h. Cells and culture supernatants were harvested to analyze PRRSV N protein expression levels (**a**) and viral production (**b**). Each value represents the mean ± SD from three independent experiments (*, *p* < 0.05; **, *p* < 0.01; ***, *p* < 0.001; ND means non-detectable)

After PRRSV attachment to Marc-145 cells, the next step of infection would be clathrin-mediated internalization [21]. Therefore, we examined virus endocytosis by treating Marc-145 cells with 5–15 μM curcumin. N protein expression decreased in a dose-dependent manner, demonstrating prevention of virus internalization (Fig. 5a). Moreover, N protein levels were reduced by factors of 0.65 to 0.02 of untreated controls, while PRRSV progeny titers deceased by 0.63 to 1.65 log compared to PRRSV infection without curcumin (Fig. 5b). Therefore, curcumin inhibits PRRSV internalization by Marc-145 cells.

### Curcumin inhibits PRRSV uncoating by blocking virus-mediated cell fusion

**Fig. 6:**
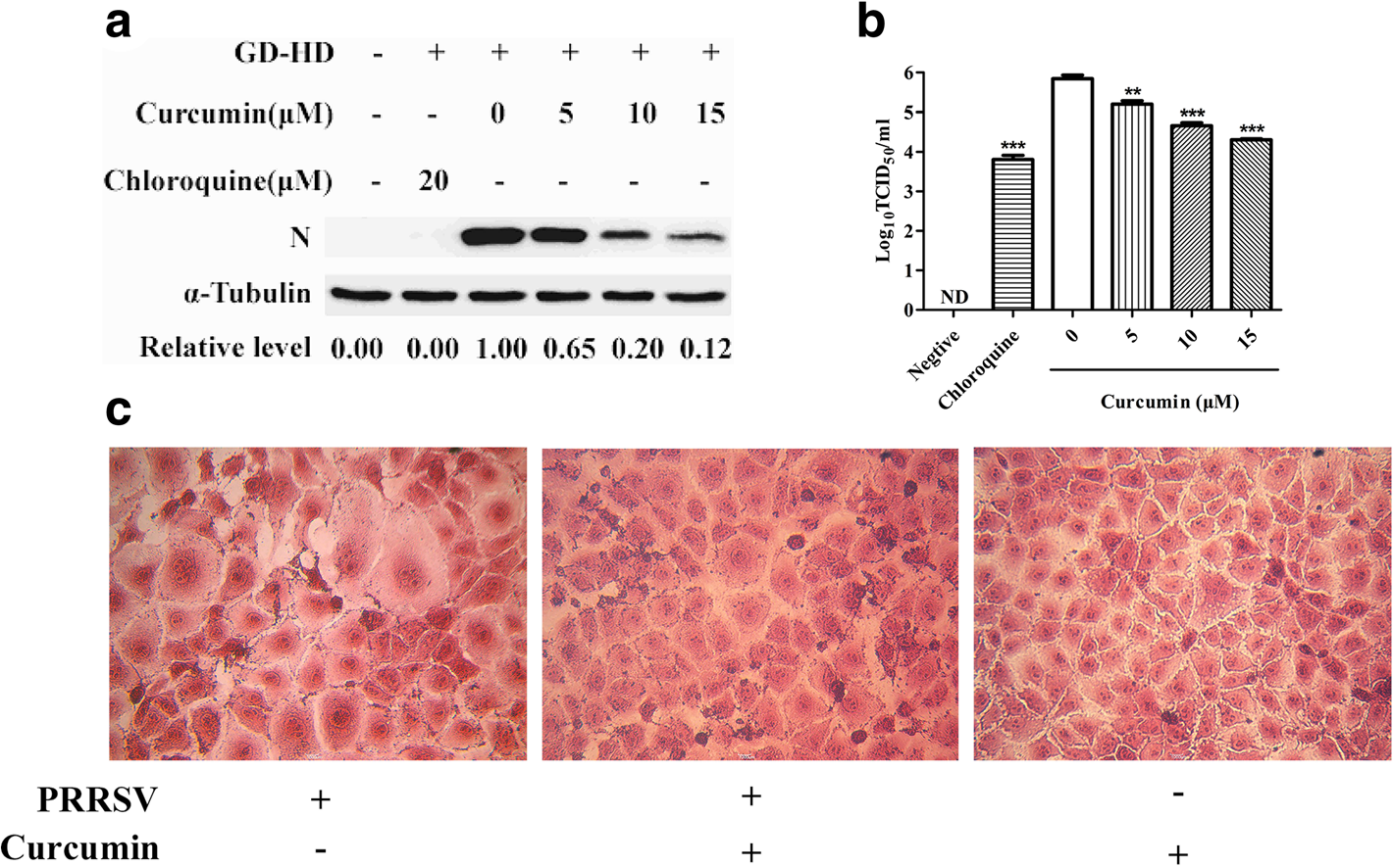
Assessment of the effect of curcumin on post-internalization and cell fusion in PRRSV-infected Marc-145 cells. Marc-145 cells were infected with GD-HD (MOI: 0.1) and subjected to a virus post-internalization assay. PRRSV N protein levels (**a**) and virus titers in cell supernatants (**b**) were detected using western blot and CCID_50_ assay, respectively. Each value represents the mean ± SD from three independent experiments (*, *p* < 0.05; **, *p* < 0.01; ***, *p* < 0.001; ND means non-detectable). For the observation of cell fusion, Marc-145 cells were subjected to the low pH-dependent cell fusion assay. Cell changes were observed under light microscope (**c**)

After internalization and transport of PRRSV particles to endosomes, virus glycoprotein conformational changes occur and virus membranes fuse with endosome membranes, resulting in uncoating of virus particles [24]. To establish if curcumin affects PRRSV uncoating in Marc-145 cells, post-internalization and low pH-dependent cell fusion assays were conducted. Treatment using 5–15 μM curcumin led to a dose-dependent reduction in N protein levels (Fig. 6a). Furthermore, a dose-dependent increase in curcumin concentration correlated with reduced PRRSV progeny titers (Fig. 6b). Therefore, curcumin inhibited post-internalization events of PRRSV infection of Marc-145 cells, including virus uncoating. Additionally, in the low pH-dependent cell fusion assay, curcumin markedly blocked cell fusion (Fig. 6c) with subsequent inhibition of virus uncoating.

### Curcumin inhibition of PRRSV infection in PAMs

**Fig. 7:**
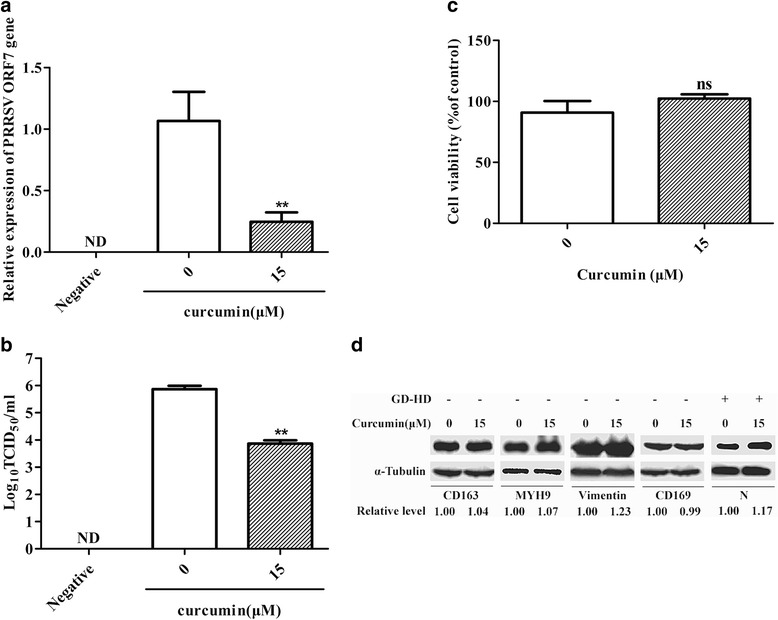
Evaluation of the effect of curcumin on PRRSV entry and levels of receptors in PAMs. Treatment of PAMs was performed as in Fig. 1. After incubation for 36 h, total cellular RNA was extracted and PRRSV ORF7 RNA levels were detected by qRT-PCR (**a**). Culture supernatants were tested for virus titers (**b**). Curcumin cytotoxicity was detected in PAMs using a CCK8 assay and is presented as experimental cell viability relative to cell viability of untreated control (100%) (**c**). PAMs were incubated with curcumin (15 μM) at 37 °C for 1 h and CD163, MYH9, vimentin, and CD169 protein levels were detected by western blot. In addition, PAMs were incubated with curcumin at 37 °C for 1 h and then the cells were infected with PRRSV GD-HD strain (MOI: 0.1). After 36 h, PRRSV N protein expression levels were determined by western blot (**d**). Each value represents the mean ± SD from three independent experiments (*, *p* < 0.05; **, *p* < 0.01; ***, *p* < 0.001; ND means non-detectable)

In addition to cell lines, to evaluate whether curcumin inhibits infection of primary cells, we isolated PAMs and tested these cells as conducted for Marc-145 cells above. As expected, 15 μM curcumin blocked PRRSV infection in PAMs and reduced ORF7 mRNA levels (Fig. 7a) and virus production (a 2.02 log decrease) (Fig. 7b). Additionally, CCK-8 assays ruled out curcumin cytotoxic effects as the cause of inhibition of viral infection (Fig. 7c).

To determine whether curcumin inhibition of PRRSV infection was due to downregulation of the known major PRRSV receptors, the expression levels of CD163, MYH9, vimentin and sialoadhesin (CD169) [25] were detected after 1 h of curcumin treatment of PAMs. The results in Fig. 7d showed that curcumin did not downregulate the expression levels of these receptors. Moreover, this treatment had no effect on PRRSV infection in PAMs (Fig. 7d).

## Discussion

PRRSV remains a major threat to the swine industry worldwide because effective measures for preventing and treating PRRS do not yet exist. Therefore, anti-PRRSV drugs are urgently needed to eradicate PRRS. In this study, we studied the mechanism of action of curcumin underlying inhibition of PRRSV infection.

Curcumin is a broad-spectrum antiviral agent, it inhibits entry of lentiviral vectors pseudotyped with HCV and CHIKV envelope proteins into hepatocytes and 293 T cells, respectively [13, 15]. Because our data suggest that curcumin inhibits different strains of genotype 2 PRRSV infection in Marc-145 (Figs. 1, 2) and PAMs (Fig. 7a, b), we systematically elucidated curcumin’s antiviral mechanism of action.

To investigate curcumin inhibition of PRRSV infection, the effect of curcumin on virus binding was first tested. Our results showed that expression levels of major PRRSV receptors (Figs. 3a-c and 7d) and susceptibility of target cells to virus (Figs. 3d, e and 7d) were unaffected by curcumin. In contrast, after incubation directly with virus at 37 °C, curcumin inhibited PRRSV attachment to Marc-145 cells (Fig. 4b), suggesting that curcumin reduced the infectivity of PRRSV particles. It has been reported that curcumin could affect membrane fluidity [26]. A previous study found that curcumin reduced the infectivity of HCV particles through decreasing the fluidity of viral envelopes, not rupturing virus particles [13]. PRRSV particles are comprised of an isometric core surrounded by a lipid-containing envelope. Moreover, our data showed that curcumin could inhibit PRRSV-mediated cell fusion (Fig. 6c). We therefore speculate that curcumin attenuated the infectivity of PRRSV by affecting the membrane fluidity of viral envelopes, not inactivating virus particles. After virus binding, curcumin also inhibited subsequent infection steps, virus internalization (Fig. 5) and uncoating (Fig. 6), in agreement with another study, where curcumin inhibited HCV binding and fusion [13].

The in vivo effect of curcumin has been studied in many animal disease models using multiple delivery strategies [27], but no studies of curcumin effects on PRRSV infection in vivo have been reported. One study investigated curcumin’s antiviral role against HCV in primary human hepatocytes in vitro, demonstrating that the α,β-unsaturated ketone groups of curcumin were largely responsible for antiviral activity [13]. Another study reported that 200 mg/kg dietary curcumin had no effect on animal well-being or immune status [28]. Thus, curcumin shows promise as an antiviral agent and is well tolerated in pigs with minimal detrimental effects.

## Conclusions

In summary, this research demonstrated the effect of curcumin, a natural compound, to prevent infection by several PRRSV strains. Curcumin affected PRRSV internalization and uncoating during the early virus infection stage and shows promise as an inhibitor of PRRSV infection in vitro. Further studies are needed to evaluate curcumin for prevention of PRRSV infection in vivo.

## Abbreviations

ANOVA: Analysis of variance
CCID_50_: 50% cell culture infection dose
CCK8: Cell Counting Kit-8
CHIKV: Chikungunya virus
DMEM: Dulbecco’s modified Eagle’s medium
HCV: Hepatitis C virus
HP-PRRSV: Highly pathogenic PRRSV
MOI: Multiplicity of infection
MYH9: Non-muscle myosin heavy chain 9
PAMs: Porcine alveolar macrophages
PRRS: Porcine reproductive and respiratory syndrome
PRRSV: Porcine reproductive and respiratory syndrome virus
qRT-PCR: Quantitative reverse transcription-PCR
VSV: Vesicular stomatitis virus
